# Supplementary figures and images for: Genome-wide circadian gating of a cold temperature response in bread wheat
Source: PLoS Genet. 2023 Sep 18;19(9):e1010947. doi: 10.1371/journal.pgen.1010947 (PMC10538658; doi:10.1371/journal.pgen.1010947)

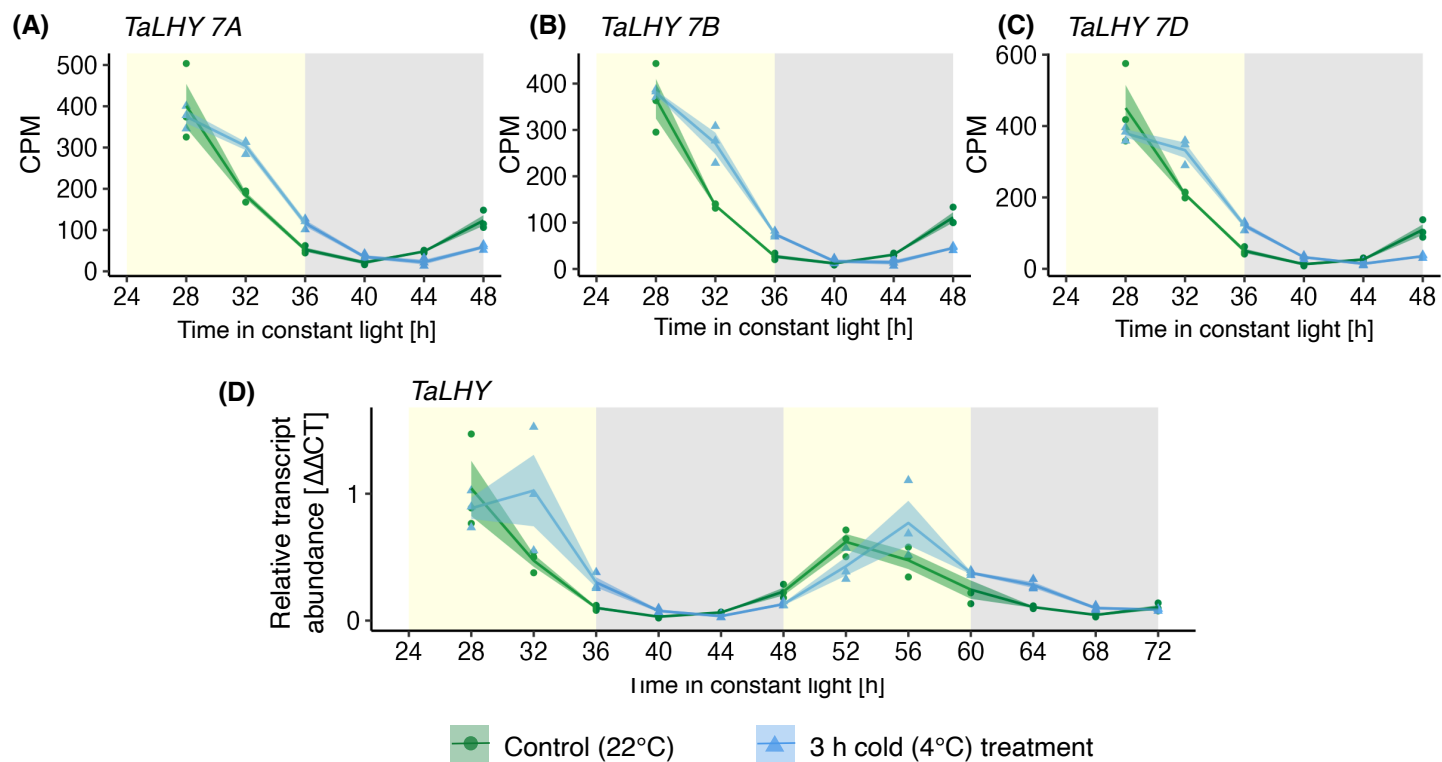

Supplement: S1 Fig — (A-C) Relative abundance of TaLHY homoeologs from the A, B and D wheat subgenomes quantified using RNA sequencing analysis. (D) Relative abundance of TaLHY transcript abundance (using primers binding all homoeologs) using RT-qPCR analysis. Solid lines are mean (N = 3). Blue/green shading = ± s.e.m. Yellow/grey shading = subjective day/night. (PDF) [file pgen.1010947.s001.pdf]

(A)

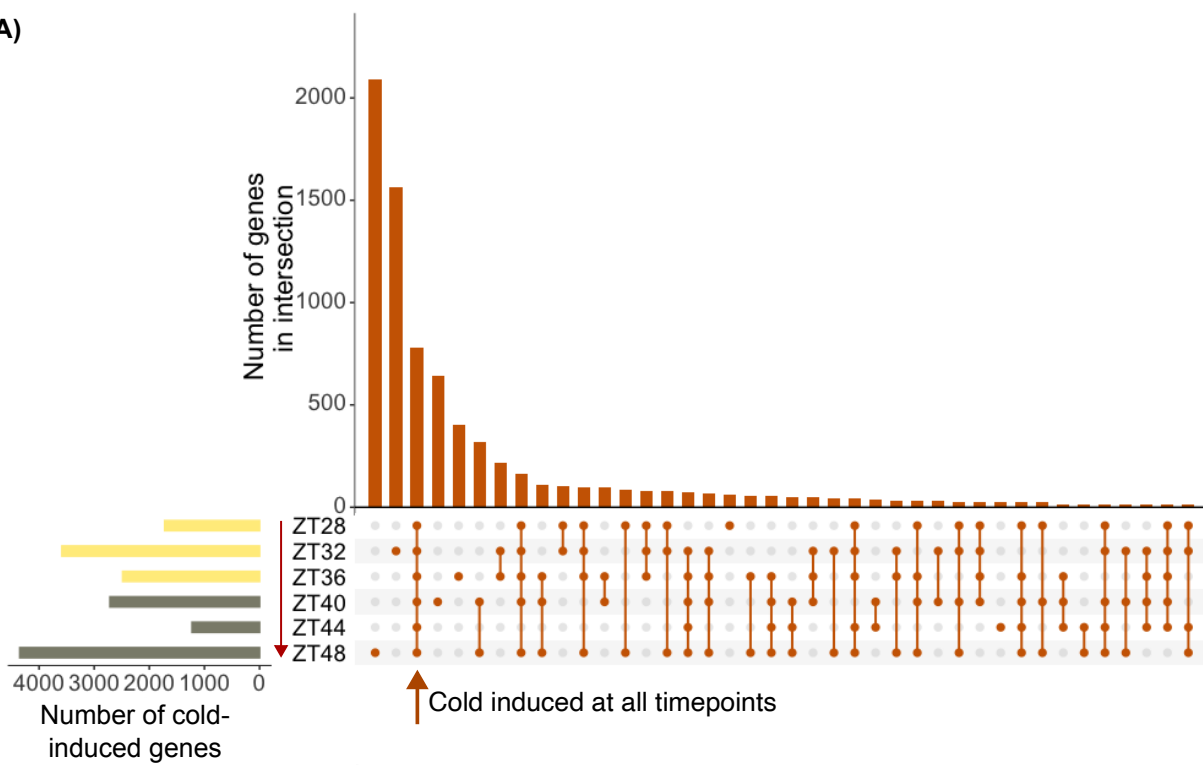

(B)

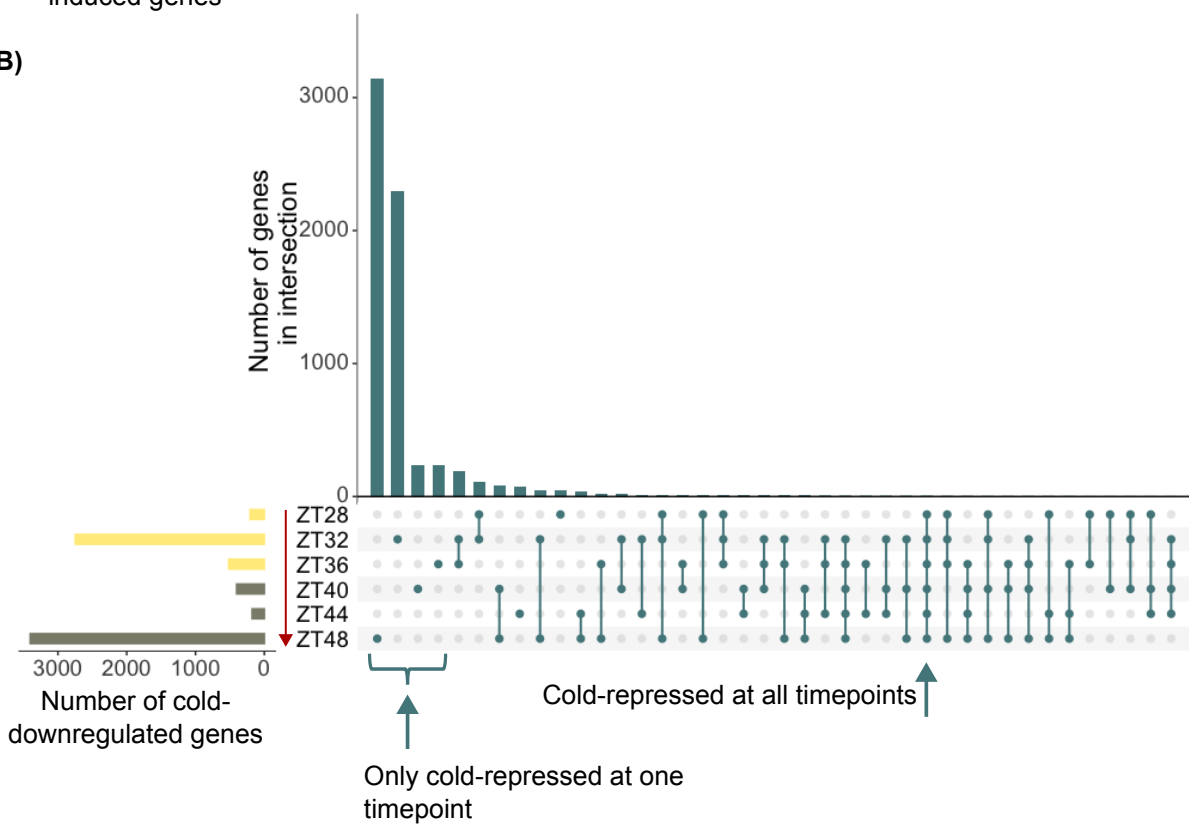

Supplement: S2 Fig — Upset plots visualising the size of the intersections between the groups of genes (A) up-regulated, and (B) down-regulated by an acute cold treatment each timepoint. In a similar manner to Venn diagrams, filled circles signify the timepoint of interest and the links between filled circles signify the intersection of interest, the size of which is reported by the height of the bar immediately above. Set size represents the total amount of DEGs detected at each timepoint. (PDF) [file pgen.1010947.s002.pdf]

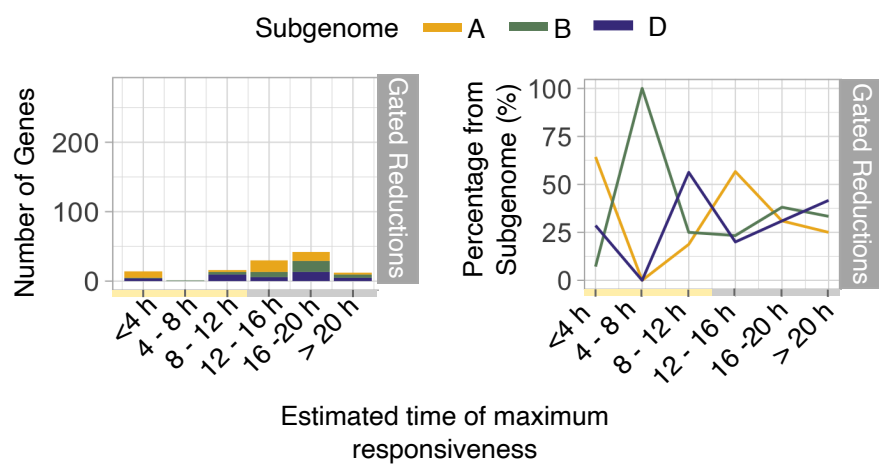

Supplement: S3 Fig — Small group sizes for each Estimated Time of Maximum Responsiveness group resulted in large variation in the proportion of transcripts derived from each subgenome. (PDF) [file pgen.1010947.s003.pdf]

Estimated time of maximum responsiveness (ETMR) group

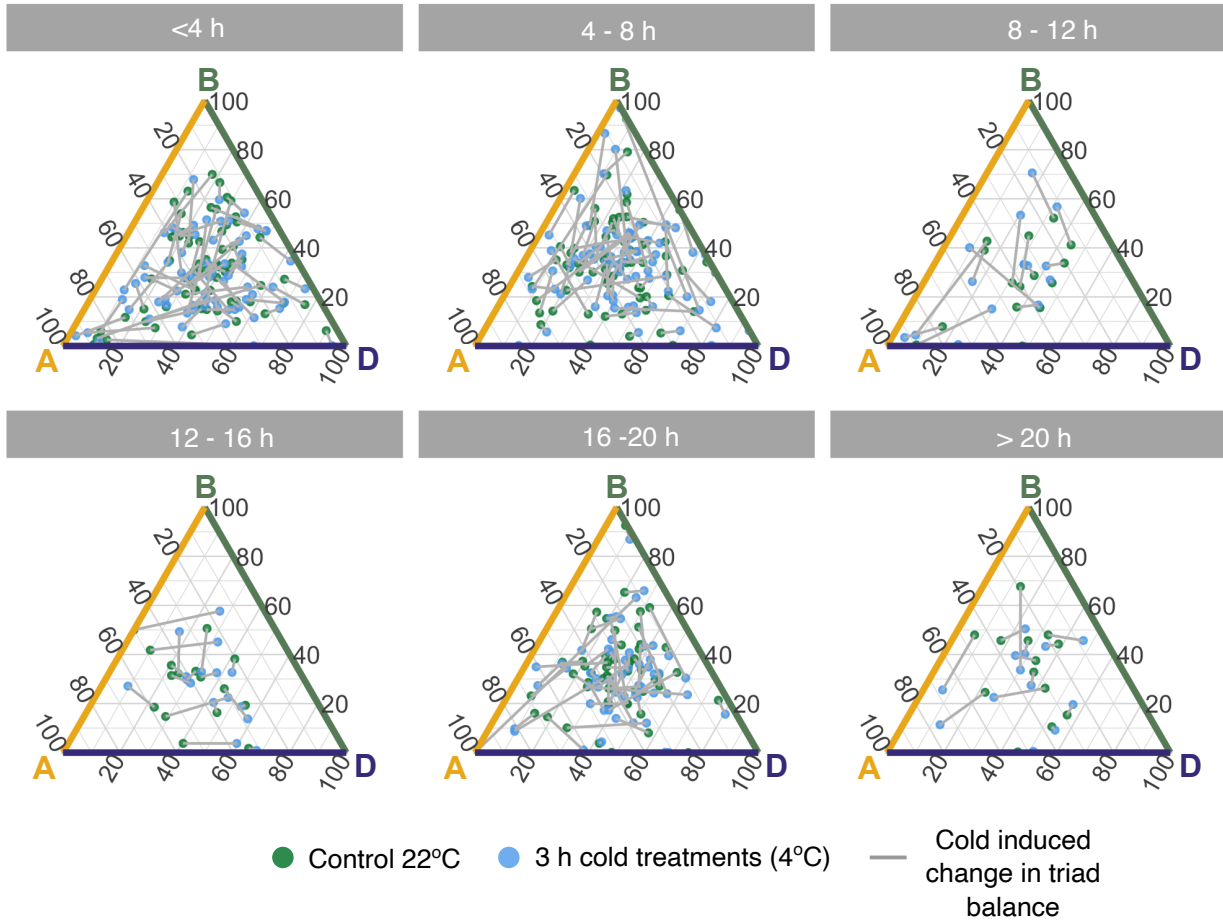

Supplement: S4 Fig — The relative contributions of homoeologs to total triad transcript abundance following each cold treatment and in its respective control, split by the estimated time of maximum responsiveness (ETMR) groups. Ternary plots as described in Fig 5C–5E and cold treatment data are those presented in Fig 5F. (PDF) [file pgen.1010947.s004.pdf]

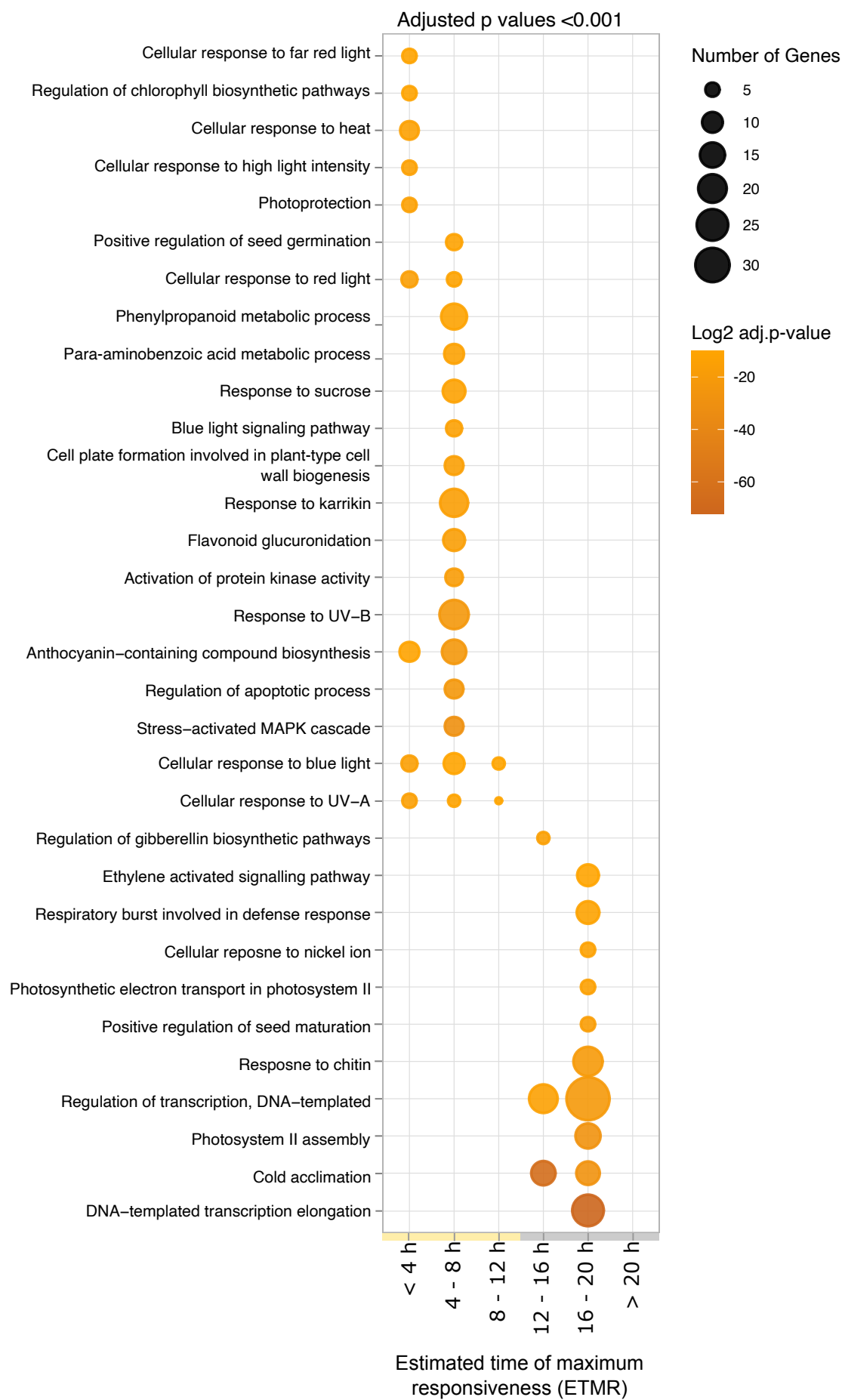

Supplement: S5 Fig — GO term enrichment within each estimated time of maximum responsive cold upregulation. Circle size represents the number of transcripts associated with the GO term, and circle colour indicates the Benjamini-Hochberg adjusted weighted Fisher p-value. (PDF) [file pgen.1010947.s005.pdf]

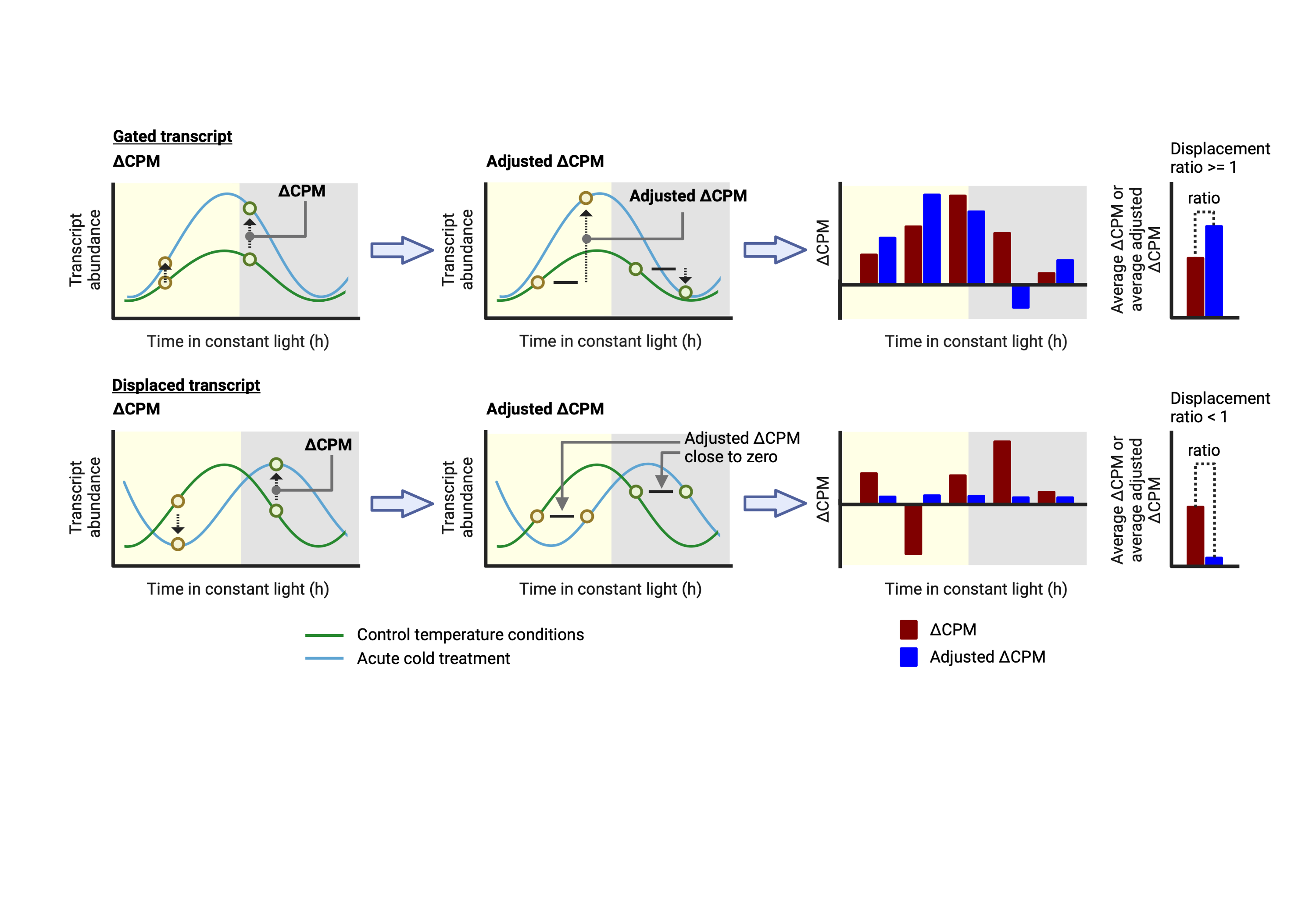

Supplement: S6 Fig — The top row of diagrams shows a hypothetical transcript that has a circadian gated response to cold, with the phase of the gate aligned with the phase of the oscillation under control temperature conditions. The lower row of diagrams shows a hypothetical transcript that has a displaced response to cold. Under these circumstances, the CPM at any given timepoint is similar to the CPM of the cold-treated sample at the subsequent timepoint, because the transcript level changes little during the cold treatment. This feature is identified from the ratio of the average ∆CPM to the average adjusted ∆CPM. (PNG) [file pgen.1010947.s006.png]

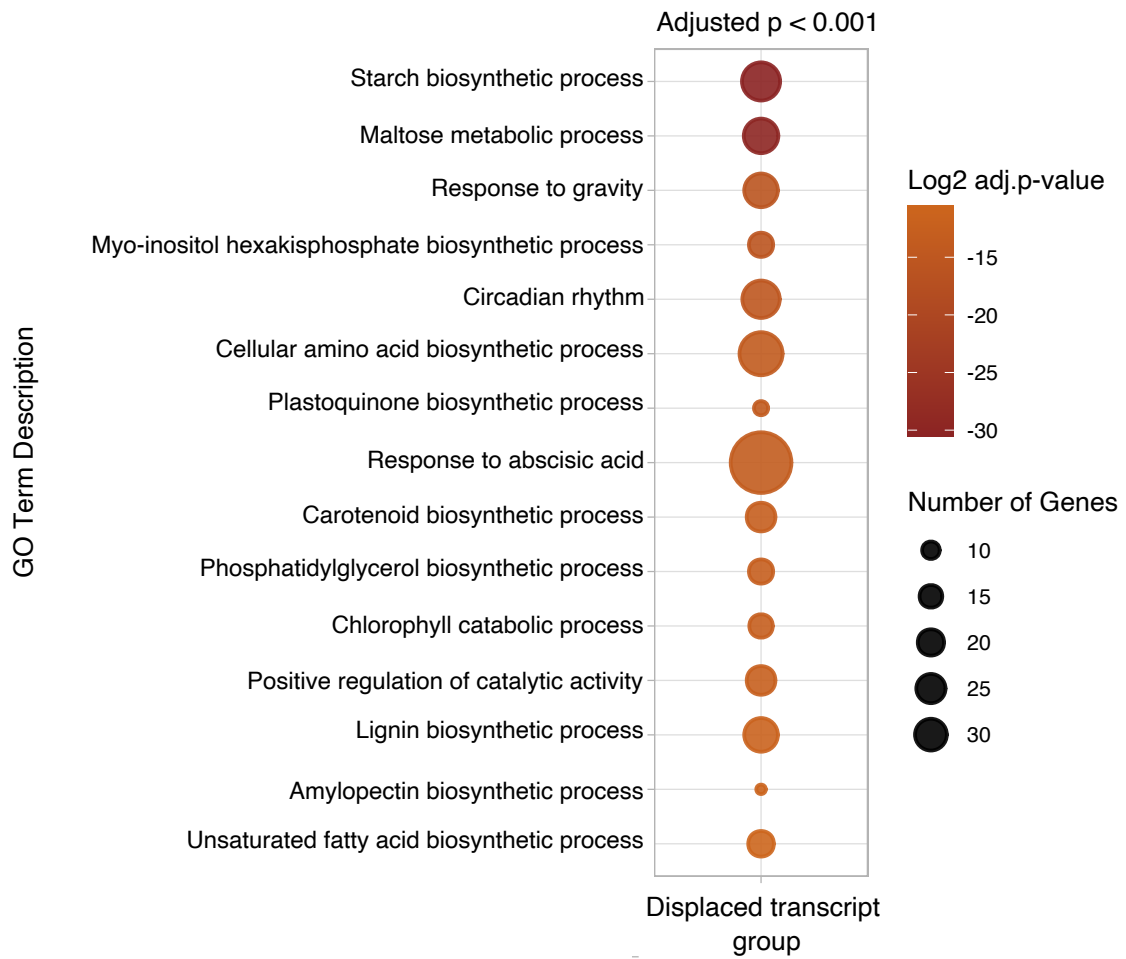

Supplement: S7 Fig — GO term enrichment for transcripts with a displacement ratio < 0.5 in response to cold, in combination with either rhythmic CPM profiles or rhythmic ∆CPM (meta3d p <0.05). Circle size represents the number of transcripts associated with the GO term, and circle colour indicates the Benjamini-Hochberg adjusted weighted Fisher p-value (adjusted p < 0.001). (PDF) [file pgen.1010947.s007.pdf]

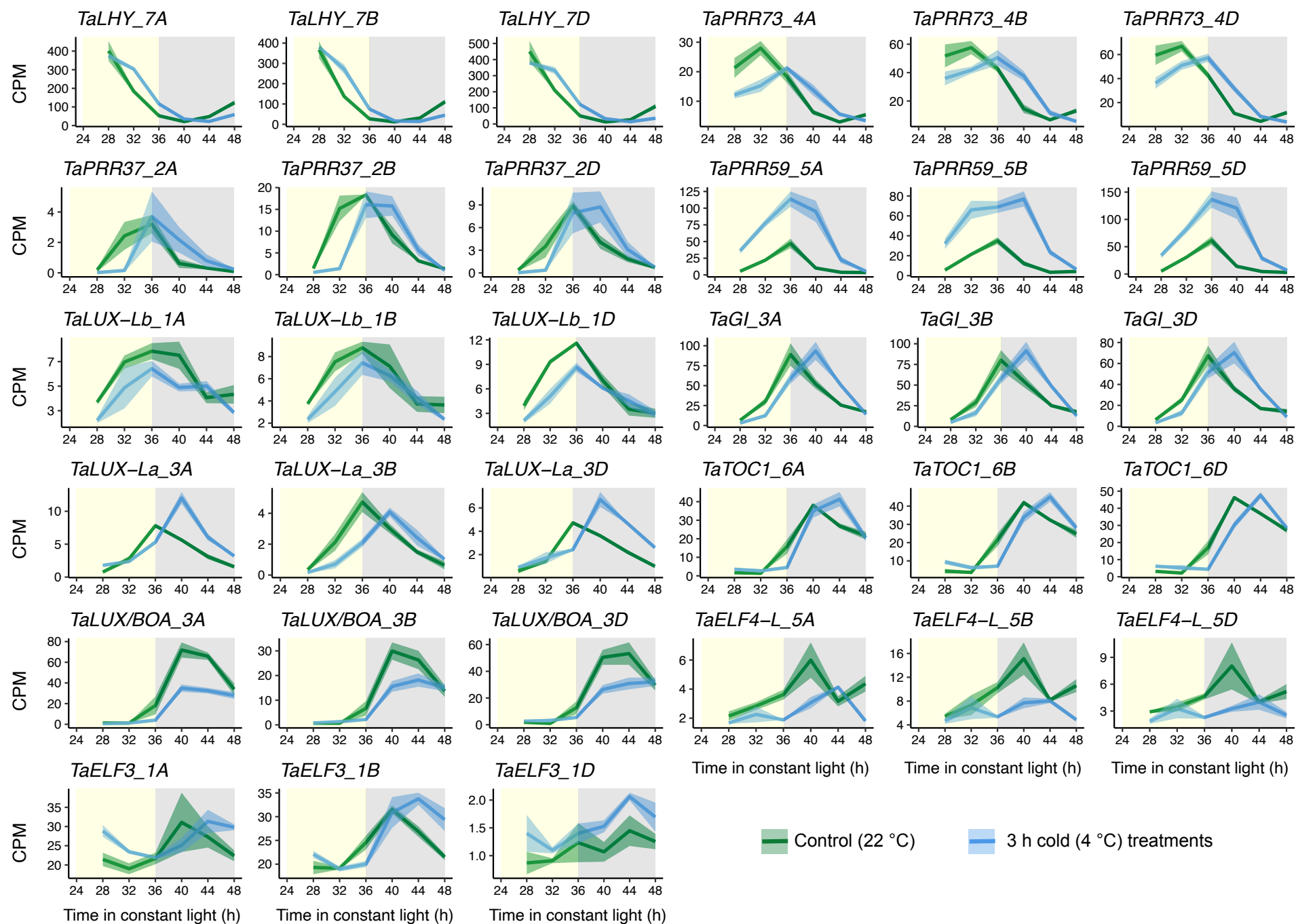

Supplement: S8 Fig — Yellow/grey shading = subjective day/night. Solid lines are mean (N = 3 biological replicates). Individual data points omitted for clarity within multi-panel figure. Blue/green shading = ± s.e.m. (PDF) [file pgen.1010947.s008.pdf]

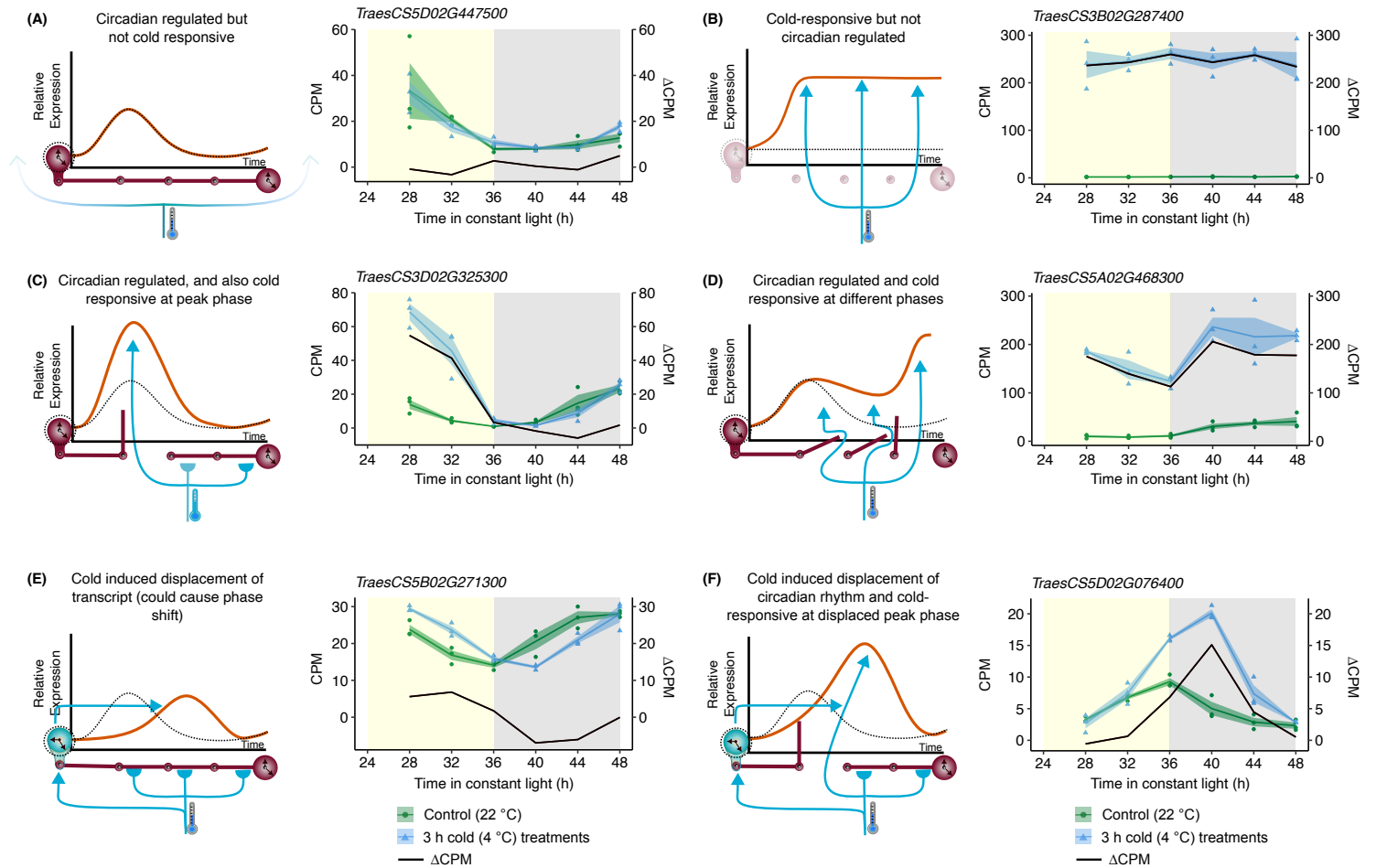

Supplement: S9 Fig — In this figure, the regulatory concepts from Fig 7 are shown alongside example transcripts. Transcripts (A) TraesCS5D02G447500, (B) TraesCS3B02G287400, (C) TraesCS3D02G325300, (D) TraesCS5A02G468300, (E) TraesCS5B02G271300, (F) TraesCS5D02G076400. Yellow/grey shading = subjective day/night. Solid lines are mean (N = 3 biological replicates). Blue/green shading = ± s.e.m. On plots of transcript data, black line represents ∆CPM. (PDF) [file pgen.1010947.s009.pdf]

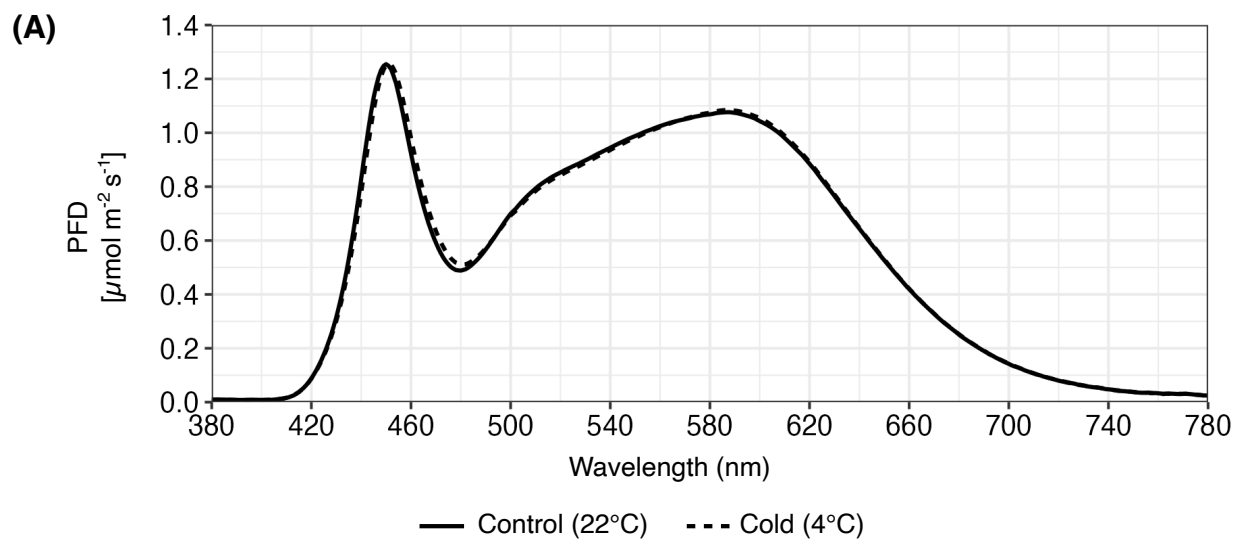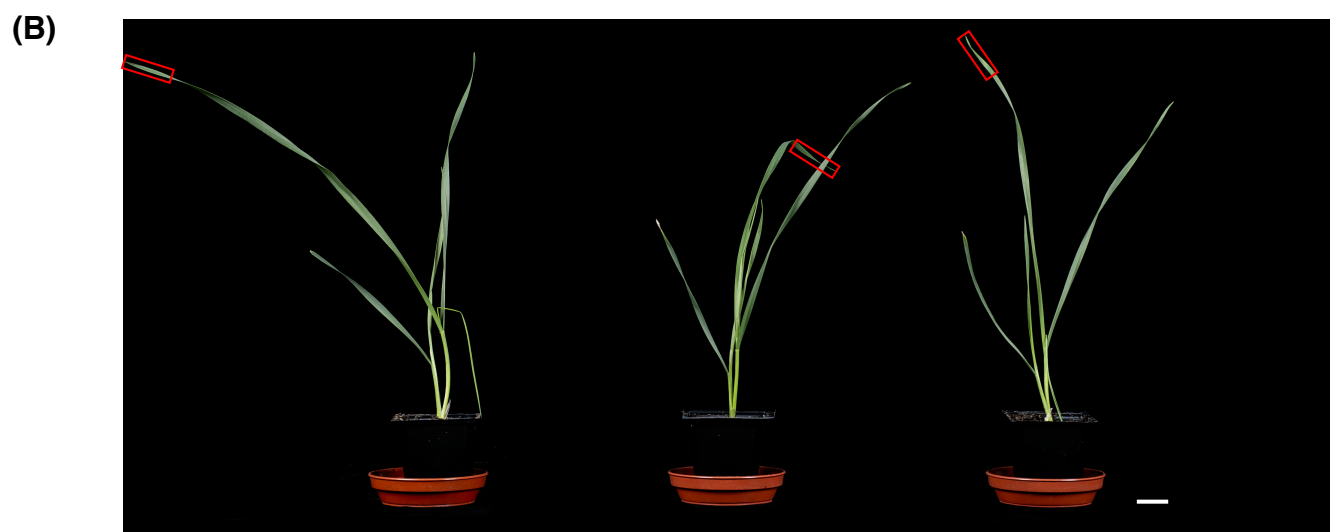

Supplement: S10 Fig — (A) Light spectra used for plant cultivation and experimentation, for control and cold temperature conditions. (B) Representative images of bread wheat seedlings after germination on damp filter paper, followed by 12 days growth on compost under 12 h: 12 h light dark cycles (22°C) followed by 24 h of constant light. Seedlings were equivalent to Zadok Stage GS1.2 at time of sampling. Red boxes indicate region of second leaf that was sampled. Scale bar 20 mm. (PDF) [file pgen.1010947.s010.pdf]

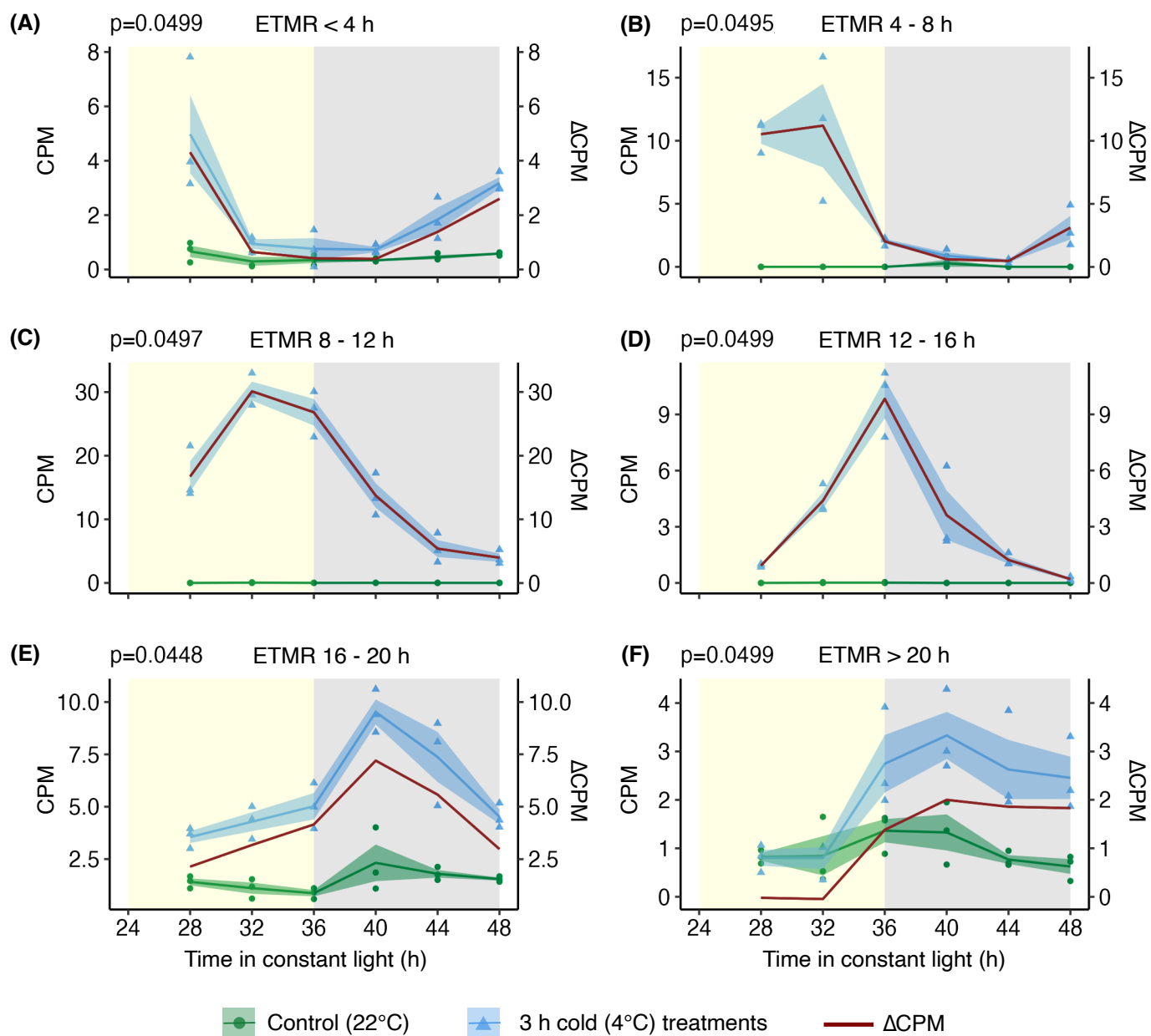

Supplement: S11 Fig — Example transcripts that have rhythmic ∆CPM (meta2d p <0.05) and p-value close to the cut-off limit, demonstrating the level of statistical stringency was appropriate for detection of circadian-gated transcripts. Transcripts are from ETMR grouping (A) < 4 h, TraesCS1A02G210400, (B) 4–8 h, TraesCS5D02G343300, (C) 8–12 h, TraesCS5D02G318100, (D) 12–16 h, TraesCS5A02G311100, (E) 16–20 h, TraesCS3B02G342936, (F) > 20 h, TraesCSU02G072000. Yellow/grey shading = subjective day/night. Solid lines are mean (N = 3 biological replicates). Blue/green shading = ± s.e.m. Red line represents ∆CPM. (PDF) [file pgen.1010947.s011.pdf]
